# Supplementary material for: Identification of photoperiod-induced specific miRNAs in the adrenal glands of Sunite sheep (Ovis aries)
Source: Front Vet Sci. 2022 Jul 22;9:888207. doi: 10.3389/fvets.2022.888207 (PMC9354845; doi:10.3389/fvets.2022.888207)
Supplement: Supplementary file 1 [file Data_Sheet_1.ZIP › Supplementary file/miRDeep2 file.zip!/miRDeep2 file/Know_Structure/LP42A3/oar-mir-218a.pdf]

miRBase precursor : oar-mir-218a
Total read count : 73282
oar-miR-218a read count : 73282
remaining reads : 0

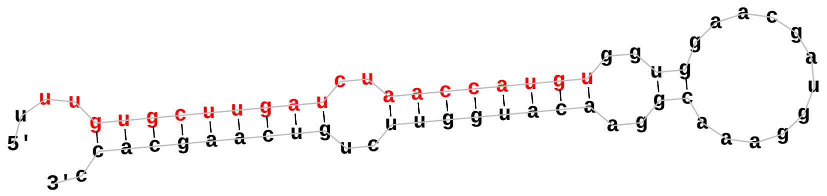

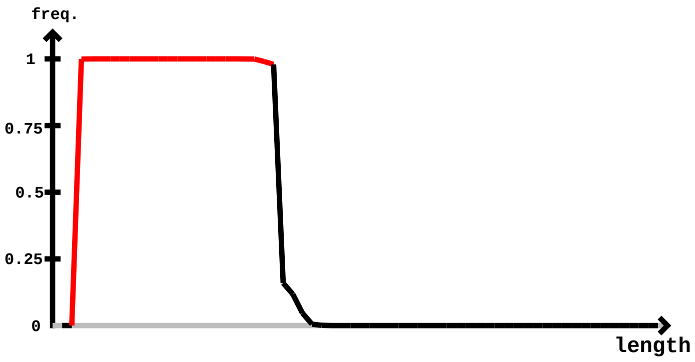

oar-miR-218a

```
5'- uuugugcuugaucuaaccauguggguggaacgauggaaacggaacaugguucugucaagcacc   -3'   exp
    ...(((((((((..((((((((..((............))..))))))))..))))))))).   reads    mm    sample
    uuugugcuugaucuaaccaug..........................................   1        0     seq
    uuugugcuugaucuaaccaugu.........................................   10       0     seq
    Guugugcuugaucuaaccaugug........................................   1        1     seq
    .uugAgcuugaucuaacc.............................................   1        1     seq
    .uugugcuugaucuaacc.............................................   16       0     seq
    .uugugcuugaucuaacca............................................   29       0     seq
    .uugugcuugaucuaaccau...........................................   657      0     seq
    .uugugcuugaucuaacAau...........................................   3        1     seq
    .uugugcuugaucuaaccaug..........................................   807      0     seq
    .uugugcuugaucuaacAaugu.........................................   156      1     seq
    .uugugcuugaucuaaccaugu.........................................   59906    0     seq
    .uugugcuugaucuaaccaugug........................................   3046     0     seq
    .uugugcuugaucuaaccauguggg......................................   5173     0     seq
    .uugugcuugaucuaaccauguggu......................................   3042     0     seq
    .uugugcuugaucuaaccauguggug.....................................   6        0     seq
    .uugugcuugaucuaaccaugugguU.....................................   308      1     seq
    .uugugcuugaucuaaccaugugguUg....................................   69       1     seq
    ..ugugcuugaucuaaccaugu.........................................   14       0     seq
    ..ugugcuugaucuaaccaugug........................................   1        0     seq
    ..ugugcuugaucuaaccaugugg.......................................   21       0     seq
    ..ugugcuugaucuaaccauguggu......................................   9        0     seq
    ..ugugcuugaucuaaccaugugguU.....................................   2        1     seq
    ...gugcuugaucuaaccaugu.........................................   2        0     seq
    ....ugcuugaucuaaccaugu.........................................   1        0     seq
    ....ugcuugaucuaaccaugugguUg....................................   1        1     seq
```
